# The Synthesis and Structural Characterization of Graft Copolymers Composed of γ-PGA Backbone and Oligoesters Pendant Chains

**DOI:** 10.1007/s13361-017-1731-y

**Published:** 2017-07-10

**Authors:** Iwona Kwiecień, Iza Radecka, Marek Kowalczuk, Katarzyna Jelonek, Arkadiusz Orchel, Grażyna Adamus

**Affiliations:** 1 Centre of Polymer and Carbon Materials, Polish Academy of Sciences, M. Curie-Skłodowskiej 34 Street, 41-819 Zabrze, Poland; 2 School of Biology, Chemistry, and Forensic Science, Faculty of Science and Engineering, University of Wolverhampton, Wulfruna Street, Wolverhampton, WV1 1SB UK; 3 School of Pharmacy with the Division of Laboratory Medicine in Sosnowiec, Medical University of Silesia in Katowice, Chair and Department of Biopharmacy, 8 Jednosci Street, Sosnowiec, 41-208 Poland

**Keywords:** Biopolymers, Graft copolymers, Polyamides, Polyesters, Mass spectrometry

## Abstract

The novel copolymers composed of poly-γ-glutamic acid (γ-PGA) and oligoesters have been developed. The structures of the obtained copolymers including variety of end groups were determined at the molecular level with the aid of electrospray ionization multistage mass spectrometry (ESI-MS^n^). The fragmentation experiment performed for the selected sodium adducts of the copolymers confirmed that the developed methods lead to the formation of graft copolymers composed of poly-γ-glutamic acid (γ-PGA) backbone and oligoesters pendant chains. Moreover, it was established that fragmentation of selected sodium adducts of graft copolymers proceeded via random breakage of amide bonds along the backbone and ester bonds of the oligoesters pendant chains. Considering potential applications of the synthesized copolymers in the area of biomaterials, the hydrolytic degradation under laboratory conditions and *in vitro* cytotoxicity tests were performed. The ESI-MS^n^ technique applied in this study has been proven to be a useful tool in structural studies of novel graft copolymers as well as their degradation products.

**Electronic supplementary material:**

The online version of this article (doi:10.1007/s13361-017-1731-y) contains supplementary material, which is available to authorized users.

**Graphical Abstract:**
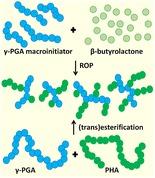
ᅟ

## Introduction

Poly-γ-glutamic acid (γ-PGA), a commercially available biopolymer made of D- or L-glutamic acid units connected by amide linkages, is produced during fermentation by various bacteria. Production of this polymer by microbial fermentation has been widely investigated and it was found that selection of bacterial strain as well as nutrient type, ionic strength, and fermentation conditions are factors that affect the enantiomeric composition and molecular mass of obtained γ-PGA [1–3]. The γ-PGA is biodegradable, nontoxic for humans, and edible; therefore it has been used in the synthesis of various materials, which were applied in wide range of fields [4, 5]. For example, the γ-PGA/chitosan composite [6] or the poly(γ-glutamic acid)-*graft*-chondroitin sulfate/polycaprolactone composite [7] were used as scaffolds in tissue engineering. The hydrogels prepared by cross-linking of γ-PGA with dihalogenoalkanes [8, 9], alkanediamine [10], or various saccharides [11] have potential application as controlled release systems. Moreover, the delivery systems based on γ-PGA have been developed. The γ-PGA-based delivery systems have been designed for various active substances, such as anticancer drug pixantrone dimaleate [12], cisplatin [13], insulin [14], protein [15], or fibroblast growth factor and heparin [16]. Effectiveness of these systems have been proven during in vitro and in vivo tests. Owing to the edibility of γ-PGA, this biopolymer could find applications in food industry, for example as a bitterness relieving agent [17], texture modifier for baked foods like wheat bread [18], or cryoprotectant for probiotic bacteria [19]. On the other hand, the γ-PGA has been successfully applied for the removal of heavy metals from wastewaters [20, 21].

Polyhydroxyalkanoates, a family of biodegradable polyesters, are produced from renewable resources (e.g., glucose) by numerous microorganisms. Use of waste and non-food competing sources as substrates is the focus of interest of recent research studies [22–24]. Synthetic polyhydroxyalkanoates can be obtained via anionic ring-opening polymerization (ROP) of β-substituted β-lactones [25–27]. Bacterial PHA, as well as synthetic PHA, have been exploited widely for various applications, including the medical field, for example as drug delivery systems [28, 29], scaffolds [30, 31], vascular systems [32, 33], or sutures [34, 35].

Taking into account the already known materials based on poly-γ-glutamic acid and their properties, the γ-PGA seems to be a promising starting compound for further modifications to obtain biomaterials with many potential applications. However, there are some difficulties associated to the derivatization of γ-PGA, mostly related to the poor solubility of this biopolymer in solvents commonly used in organic synthesis [36].We report synthetic approaches that overcome the problem of poor solubility of γ-PGA and enable to obtain derivatives of this biopolymer. However, it is noteworthy that in case of polymers with potential applications as biomaterials, it is necessary to confirm the molecular structure of obtained products. Therefore, to verify the structures of the obtained graft copolymers composed of γ-PGA backbone and oligoesters pendant chains, electrospray ionization multistage mass spectrometry (ESI-MS^n^) has been used. The ESI-MS^n^ technique has been successfully used in various polyester studies as well as in polyamides studies. The ESI-MS^n^ technique has been applied to get detailed structural information of numerous (co)polyesters, such as poly(butylene adipate-*co*-butylene terephthalate) [37], poly(2-methyl-3-hydroxyoctanoate) [26], poly(3-hydroxybutyrate-*co*-3-hydroxy-4-ethoxybutyrate) [27], or poly(3-hydroxybutyrate-*co*-3-hydroxyhexanoate) [38]. In studies related to polyamides, the ESI-MS^n^ technique was used, for example, for identification of polyamide cyclic oligomers [39], structural studies of polyamide dendrimer [40], or to probe the binding selectivity of a flexible cyclic polyamide [41]. Taking into consideration effectiveness of ESI-MS in structural studies of polyesters and polyamides, we assume that the use of this technique will provide detailed information about the structure of the graft copolymers studied as well as their degradation products.

## Experimental

### Materials

The poly(3-hydroxybutyrate-*co*-4-hydroxybutyrate) (M_n_ = 250,000 g/mol; dispersity index M_w_/M_n_ = 2.5; the 4HB unit content 8.8 mol %) was purchased from Tianjin Green Bio-Science (Tianjin, China); the high molecular weight poly-γ-glutamic acid (M_n_ = 150,000 g/mol; dispersity index M_w_/M_n_ = 2.05) and ultra-low molecular weight poly-γ-glutamic acid (M_n_ = 2000 g/mol; dispersity index M_w_/M_n_ = 1.43) were purchased from Shandong Freda Biotechnology Co., Ltd. (Shandong, China). Tetradecyltrimethylammonium bromide, 4-toluenesulfonic acid monohydrate, and [*R,S*]-β-butyrolactone were purchased from Sigma-Aldrich Chemie GmbH (Steinheim, Germany). Dimethyl sulfoxide (DMSO) and *N,N*-dimethylformamide (DMF) were purchased from POCH SA (Gliwice, Poland). Dialysis membrane Spectra/Por (MWCO 1000) was purchased from Carl Roth (Karlsruhe, Germany).

### Methods

Proton nuclear magnetic resonance (^1^H NMR) analyses were performed in CDCl_3_ on an Avance II 600 MHz Ultrashield Plus spectrometer (Bruker BioSpin GmbH, Rheinstetten, Germany). FTIR spectroscopy analysis was performed on Jasco FT-IR-6700 spectrometer (Jasco Corporation, Tokyo, Japan) using MultiLoop-MIR fiber probe (Harrick Scientific Products Inc., Pleasantville, NY. USA) connected to a FiberMate2 fiber optic coupler (Harrick Scientific Products Inc.). Electrospray mass spectrometry (ESI-MS^n^) analyses were performed in positive-ion mode on a Thermo LCQ Fleet ion-trap mass spectrometer (Thermo Fisher Scientific Inc., San Jose, CA, USA). Solutions of samples were introduced into the ESI source by continuous infusion by means of the instrument syringe pump with 10 μL/min flow rate. Spray voltage was set at 4.8 kV; capillary temperature was set at 200 °C; nitrogen was used as sheath gas; helium was used as the auxiliary gas. In ESI-MS/MS experiments, the precursor ions were isolated in the ion trap and activated by the collision.

#### Synthesis of Graft Copolymers Via “Grafting From” Method

The macroinitiators for “grafting from” method was obtained from ultra-low molecular weight poly-γ-glutamic acid and tetradecyltrimethylammonium bromide. Macroinitiator was subjected to benzylation by treatment with benzyl bromide in DMSO in order to estimate carboxylate active centers, similar to a procedure known from the literature [42]. Details of “grafting from” method are in the Supplementary Material.

#### Synthesis of Graft Copolymers Via (Trans)Esterification Reaction

The graft copolymers were obtained via (trans)esterification reaction from high molecular weight poly-γ-glutamic acid and poly(3-hydroxybutyrate-*co*-4-hydroxybutyrate) in the presence of 4-toluenesulfonic acid monohydrate. Details of (trans)esterification method are in Supplementary Material.

#### Hydrolytic Degradation of Graft Copolymers

Studies of hydrolytic degradation of graft copolymers were performed under laboratory conditions. Glass vials containing samples of graft copolymers (10 mg) and deionized water (5 cm^3^) were placed in a thermostatically controlled incubator set at 25 °C. Vials with samples were withdrawn in triplicate from the incubator after 1, 5, 10, and 20 wk; samples were analyzed using ESI-MS technique.

#### Assessment of Cytocompatibility of γ-PGA-Graft-(3HB-co-4HB) Copolymer

In vitro cytotoxicity was analyzed after an indirect contact of CCD-11Lu fibroblasts with extracts of the γ-PGA-graft-(3HB-*co*-4HB) copolymer by means of sulforhodamine B-based assay (In Vitro Toxicology Assay Kit, Sulforhodamine B-based; Sigma-Aldrich). Details are in Supplementary Material.

## Results and Discussion

### Grafting From Method

The first synthetic strategy for obtaining graft copolymers composed of γ-PGA backbone and oligoesters pendant chains was the anionic grafting of racemic β-butyrolactone on γ-PGA backbone (Grafting From, Scheme S1, Supplementary Material). This method required macroinitiators obtained from poly-γ-glutamic acid and tetradecyltrimethylammonium bromide, for which the method known from literature has been applied [43, 44]. Increasing solubility of the γ-PGA quaternary ammonium salt in comparison to sodium salt has been established [45]. The obtained γ-PGA macroinitiator with carboxylate active centers has been used in the reaction of anionic ring opening oligomerization of racemic β-butyrolactone. In order to estimate carboxylate active centers in obtained macroinitiator, the macroinitiator was subjected to benzylation by treatment with benzyl bromide in DMSO, similar to a procedure known from the literature [42]. Based on ^1^H NMR spectrum, the 50% functionalization degree was achieved. Therefore, it can be assumed that after reaction with tetradecyltrimethylammonium bromide, half of carboxylic groups of γ-PGA were transformed in the form of tetradecyltrimethylammonium salt. The modified γ-PGA could act as macroinitiator of anionic ring opening oligomerization of β-butyrolactone.

Advanced molecular characterization of γ-PGA-*graft*-3HB cooligomers was performed using electrospray ionization multistage mass spectrometry (ESI-MS^n^). The ESI-MS^n^ technique has been successfully applied by some of us in the structural studies of co-oligomers [46–49].

**Figure 1:**
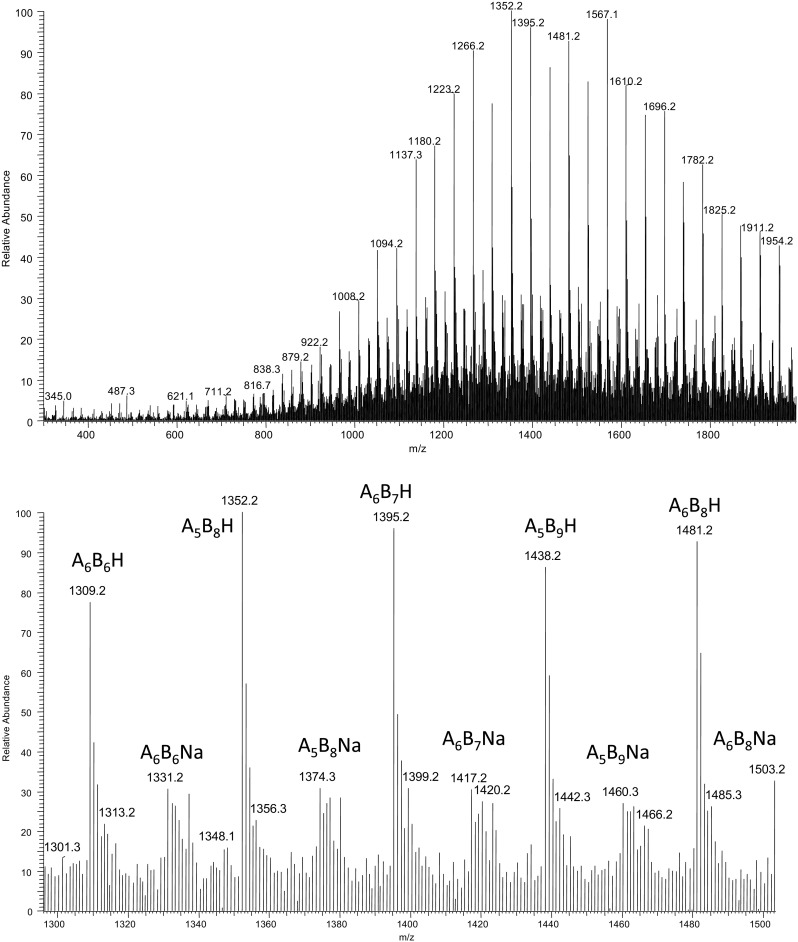
ESI-MS spectrum of γ-PGA-*graft*-3HB cooligomers and spectral expansion in the range *m/z* 1290–1505

In the ESI-MS spectrum (Figure 1) of the product obtained in the reaction of anionic ring opening oligomerization of racemic β-butyrolactone initiated by γ-PGA macroinitiator, two main series of singly charged ions corresponding to protonated or sodium adduct of γ-PGA-*graft*-(3HB) cooligomer macromolecules were visible. Moreover, an additional series corresponding to the sodium adduct of 3-hydroxybutyrate (3HB) oligomers with crotonate and carboxy end groups were also detected in lower mass range (*m/z* 500–900). However, the intensity of signals corresponding to oligo(3-hydroxybutyrate) side products has been very low.

Signals of protonated or sodiated cooligoester macromolecules in expanded mass spectrum (Figure 1) were labeled in formula A_x_B_y_H or A_x_B_y_Na, where “x” equals the number of γ-glutamate repeating units with molecular mass 129 Da, whereas “y” equals the number of 3-hydroxybutyric units (86 Da). The molecular mass of N-terminal end group and C-terminal end group in γ-PGA are 130 Da and 146 Da, respectively. Therefore, the *m/z* value for protonated cooligomer macromolecules was calculated with formula A_x_B_y_H: 130+(x-2)·129+146+y·86+1, whereas the *m/z* value for A_x_B_y_Na formula was calculated: 130+(x-2)·129+146+y·86+23. Moreover, molecular weights of three 3-hydroxybutyric units (3·86 Da) and two γ-glutamate repeating units (2·129 Da) have the same value (258 Da). Therefore, signal assignment presented in expanded mass spectrum shows only one of many possibilities.

Apart from γ-PGA-*graft*-3HB cooligomers, the 3-hydroxybutyric acid oligomers with carboxyl and crotonate end groups have been detected in lower mass range. The formation of crotonate end groups in the β-butyrolactone polymerization might be caused by a chain-transfer reaction to the monomer and/or by intermolecular carboxylate-induced α-deprotonation, which has been already reported [50, 51].

**Figure 2:**
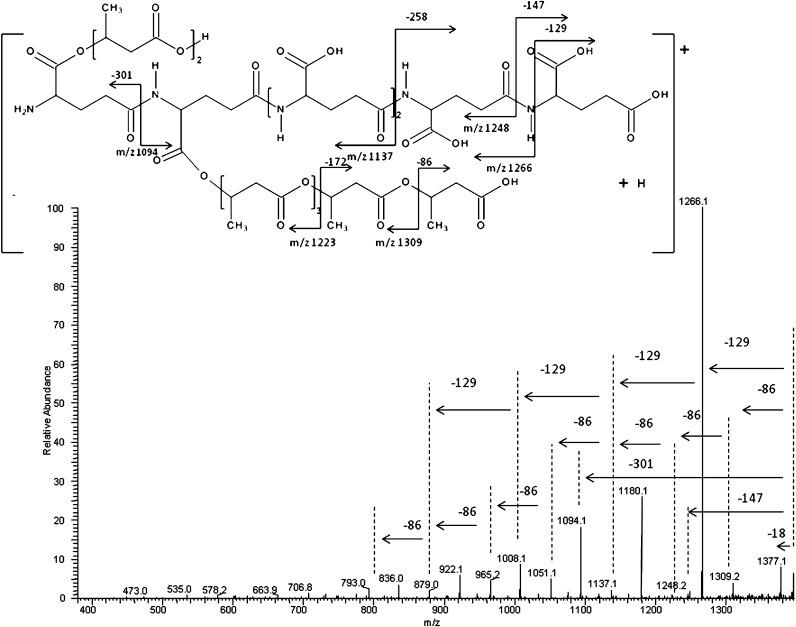
ESI-MS^2^ product ion spectrum of the sodiated γ-PGA-*graft*-3HB cooligomers at *m/z* 1395 and theoretical fragmentation pathway of one of the probable structures of this ion

To verify the structure of obtained grafted copolymers, the ESI-MS^n^ experiments were performed for selected ions visible in ESI-MS spectrum (Figure 1). Figure 2 shows the results of an ESI-MS^2^ experiment performed for the sodium adducts of co-oligomers at *m/z* 1395 (A_6_B_7_H) selected from ESI-MS spectrum of γ-PGA-graft-3HB cooligomers (Figure 1). All of the active carboxylic groups present in the γ-PGA macroinitiator should initiate anionic oligomerization of β-butyrolactone; however, oligo3-hydroxybutyrate pendant chain bonded to the γ-PGA backbone might have a different length. One of the probable structures, which consisted of six γ-glutamate repeating units and seven 3-hydroxybutyric repeating units, and the theoretical fragmentation pathway of this structure, are shown in Figure 2. The product ions at *m/z* 1309; 1223; 1137; 1051 etc. correspond to the cooligomers formed by the loss of the 3-hydroxybutyric repeating units from oligoesters pendant chains, one (86 Da), two (172 Da), three (258 Da), four (344 Da) etc. respectively. In addition, the γ-PGA backbone underwent fragmentation; for example the product ion at *m/z* 1094 corresponds to the oligomer formed by loss of the γ-glutamate repeating unit bonded with two 3-hydroxybutyric repeating units (301 Da), the product ion at *m/z* 1248 corresponds to the oligomer formed by the loss of the γ-glutamic acid (147 Da), the product ion at *m/z* 1266 corresponds to the co-oligomer formed by the loss of the pyroglutamic acid (129 Da). The product ion at *m/z* 1377 corresponds to the cooligomer formed by the loss of the water molecule (18 Da).

In order to confirm the structure of the individual cooligoester, further fragmentation experiments were performed.

**Figure 3:**
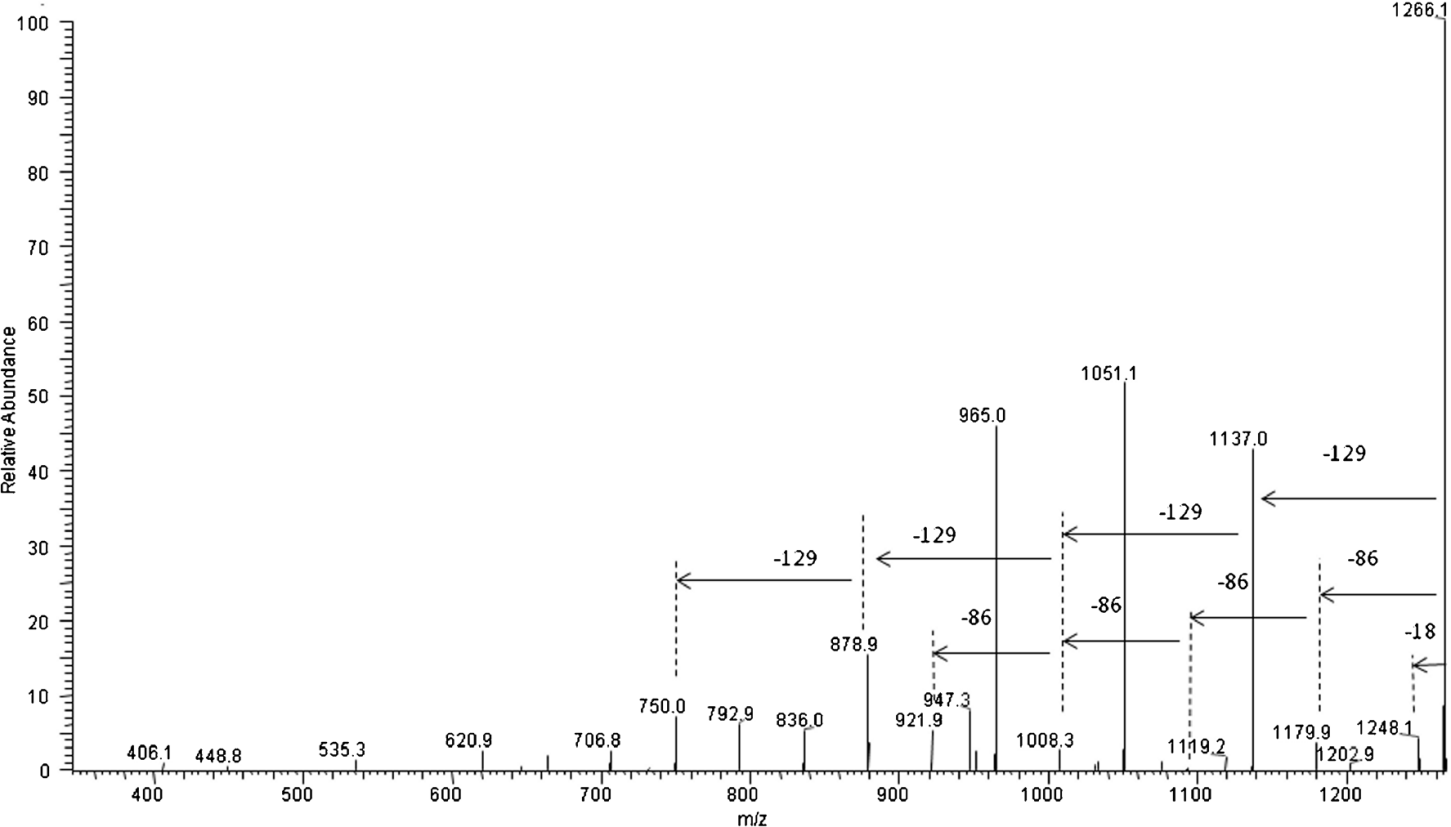
ESI-MS^3^ spectrum (in positive-ion mode) of the sodium adduct of γ-PGA-*graft*-3HB co-oligomers at *m/z* 1266 selected from the ESI-MS^2^ spectrum of γ-PGA-*graft*-3HB cooligomers at *m/z* 1395

Figure 3 shows ESI-MS^3^ spectrum of the sodium adduct of γ-PGA-*graft*-3HB cooligomers at *m/z* 1266 selected from the ESI-MS^2^ spectrum of γ-PGA-*graft*-3HB cooligomers at *m/z* 1395. The product ions at *m/z* 1180; 1094; 1008; 922 etc. correspond to the cooligomers formed by the loss of the repeating units from oligoesters pendant chains: one (86 Da), two (172 Da), three (258 Da), four (344 Da) etc., respectively. The product ions at *m/z* 1137; 1008; 879 etc. correspond to the co-oligomers formed by the loss of one, two, three etc. repeating units from γ-PGA backbone.

### Hydrolytic Degradation of γ-PGA-Graft-3HB Cooligomers

**Figure 4:**
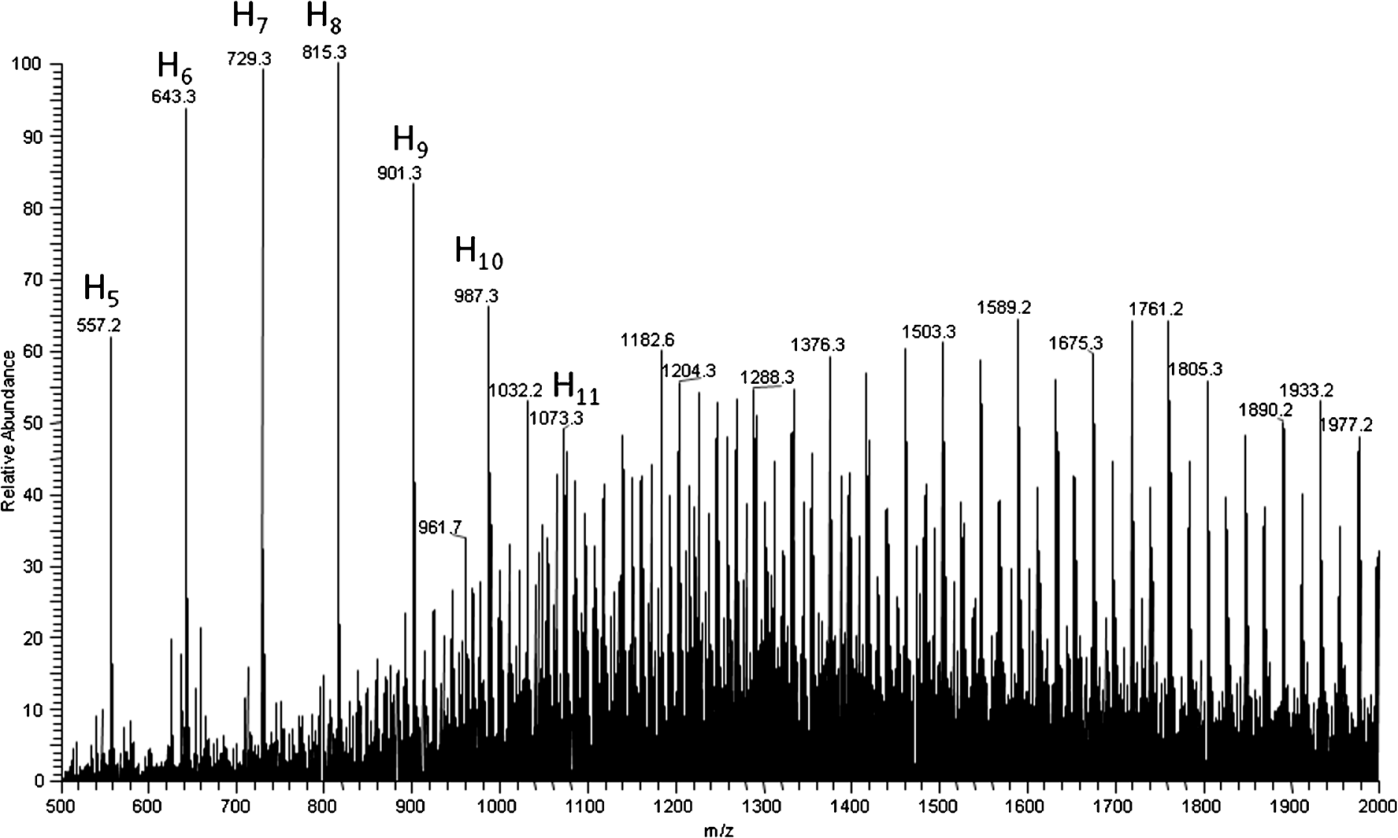
ESI-MS spectrum of γ-PGA-graft-3HB cooligomers after 20 wk of incubation in water at 25 °C

Considering the prospective application of the obtained graft copolymers as biomaterials, preliminary hydrolytic degradation studies were performed. The products of hydrolytic degradation of graft copolymers under laboratory condition were analyzed with the aid of mass spectrometry. A significant change in the distribution of the ion patterns has been observed when comparing ESI-MS spectrum of the sample after 10 wk of hydrolytic degradation (Figure 4) with ESI-MS spectrum of starting sample (Figure 1). Moreover, in Figure 4, the signals corresponding to sodium adduct of oligo(3-hydroxybutyrate) oligomers hydroxyl and carboxyl end groups appeared in mass range *m/z* 500–1100. These oligomers were formed as a product of the hydrolytic degradation of oligoesters pendant chains. Signals corresponding to sodium adduct of 3-hydroxybutyrate oligomers with hydroxyl and carboxyl end groups (Figure 4) were labeled as H_y_, where “y” equals to the number of 3-hydroxybutyric units (86 Da). The molecular mass of 3-hydroxybutyrate end group is 104 Da; therefore, the *m/z* value for H_y_ was calculated according to the formula 104+y·86+23.

During hydrolytic degradation studies, products of hydrolysis of γ-PGA backbone have not been detected. The slower hydrolytic degradation rate of oligoamide chain might have been expected because amide bonds undergo hydrolysis at considerably more vigorous conditions than ester bonds [52].

### (Trans)esterification

In further research, the second approach for obtaining the copolymers composed of γ-PGA backbone and oligoesters pendant chains based on (trans)esterification reaction has been developed. Previously, we reported (trans)esterification reaction as the method for synthesis of conjugates of model bioactive compounds with oligomer from selected biopolymers [53, 54]. In the next step of our research, it was found that the “one-pot” solvent-free synthesis enabled obtaining γ-PGA-*graft*-(3HB-*co*-4HB) cooligomers. Such graft copolymers were obtained via (trans)esterification reaction of the poly-γ-glutamic acid with poly(3-hydroxybutyrate-*co*-4-hydroxybutyrate) mediated by 4-toluenesulfonic acid monohydrate (TSA · H_2_O) carried out in the melt (Scheme S2, Supplementary Material).

**Figure 5:**
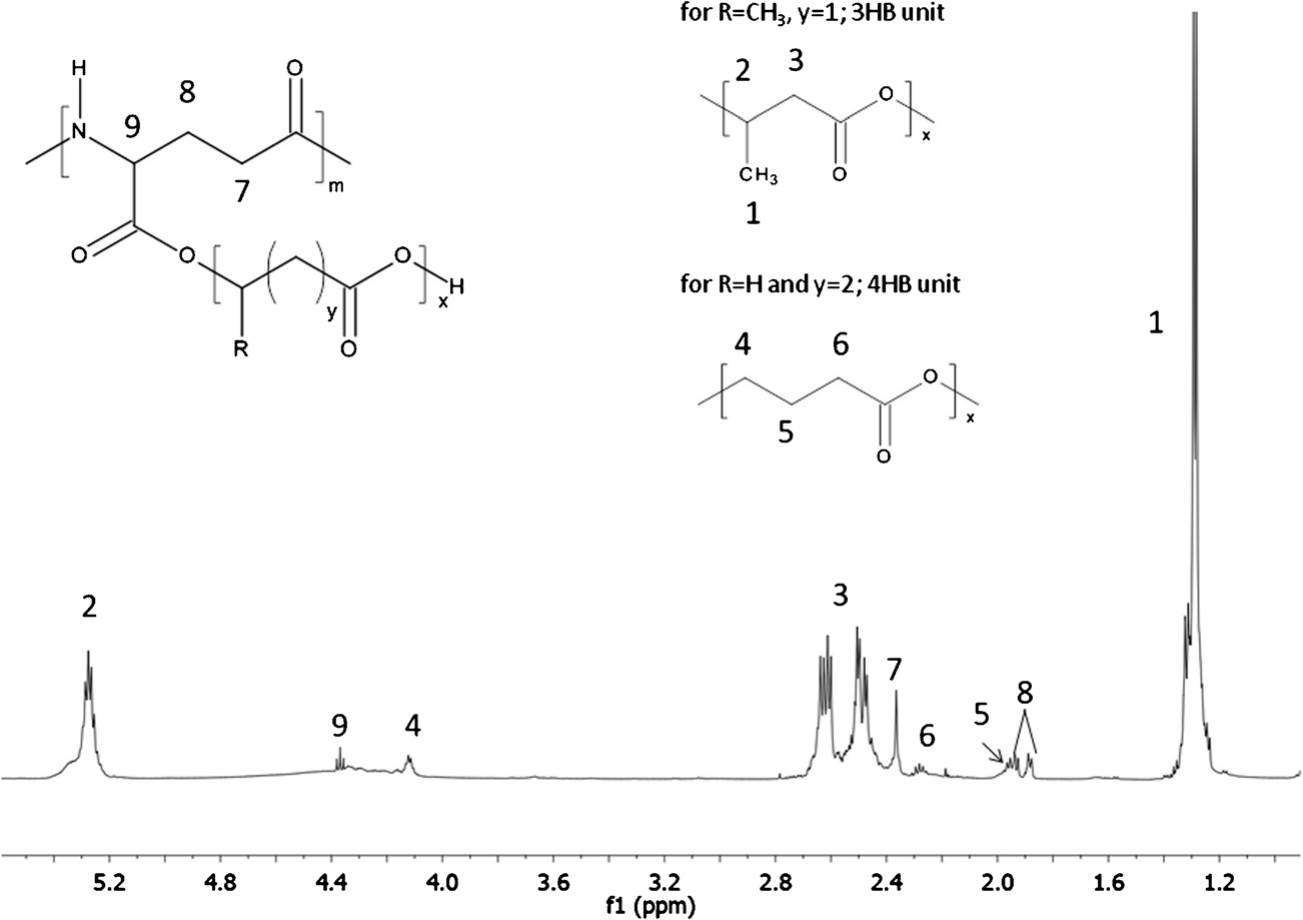
The ^1^H NMR spectrum of γ-PGA-*graft*-(3HB-*co*-4HB) cooligomers (for 3HB units R = CH_3_, y = 1, for 4HB units R = H and y = 2)

The ^1^H NMR spectrum of the products obtained through the (trans)esterification reaction poly-γ-glutamic acid with poly(3-hydroxybutyrate-*co*-4-hydroxybutyrate) in the presence of 4-toluenesulfonic acid monohydrate is presented in Figure 5. In this spectrum, signals corresponding to the protons of γ-PGA backbone (labeled 7–9) as well as signals corresponding to protons of oligo(3-hydroxybutyrate-*co*-4-hydroxybutyrate) pendant chains (labeled 1–6) were observed.

Under the (trans)esterification reaction conditions both biopolymers underwent partial hydrolysis because of the presence of water (introduced with 4-toluenesulfonic acid monohydrate). The partial thermal degradation of poly(3-hydroxybutyrate-*co*-4-hydroxybutyrate) via a random chain scission mechanism involving the β-CH hydrogen transfer at the 3-hydroxybutyrate repeating units also occurred [55]. Moreover, γ-PGA underwent partial thermal degradation via typical for polyamides mechanisms, which leads to the formation of cyclic amides [56, 57] or oligomers with unsaturated alkyl and amide end groups further transformed into nitriles [58, 59]. Owing to a variety of end groups formed via different known mechanisms, as well as the diversity of probable structure, further structural investigation was needed. For this purpose, the ESI-MS^n^ technique has been successfully applied.

**Figure 6:**
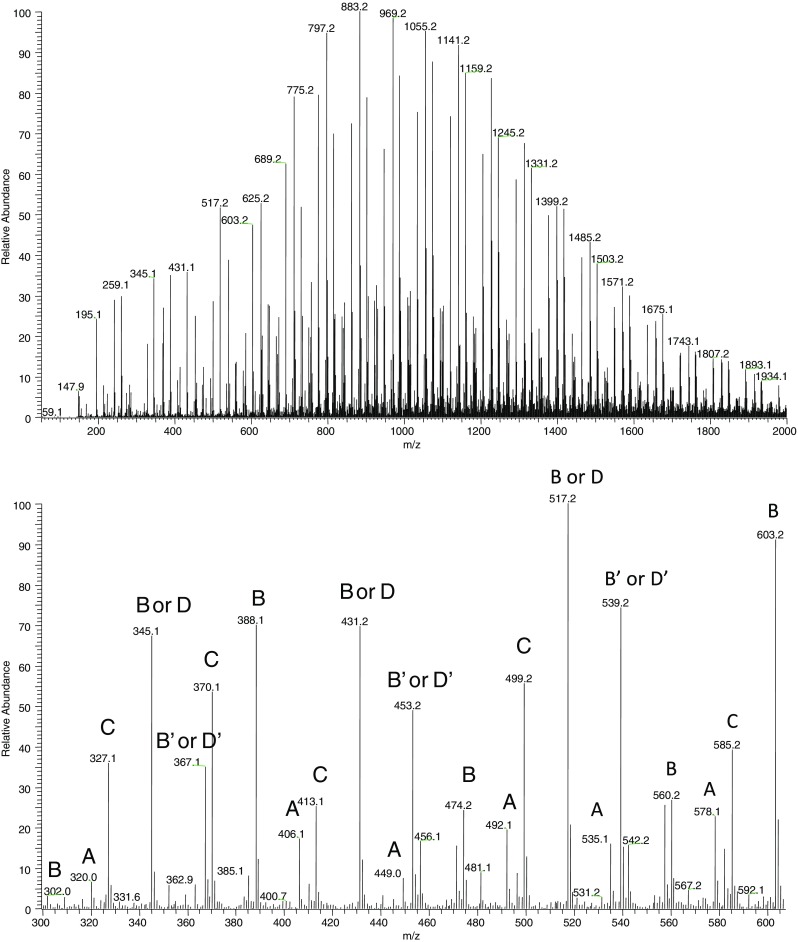
The ESI-MS of γ-PGA-*graft*-(3HB-*co*-4HB) cooligomers and spectral expansion in the range *m/z* 300–610

The structures of the ions visible in the ESI-MS spectrum in Figure 6 were assigned to the structures placed in Table 1 based on different mechanisms of thermal decomposition of polyamides discussed in literature [56–59]. Signals in ESI-MS spectrum correspond to sodium or proton adducts of γ-PGA-*graft*-(3HB-*co*-4HB) cooligomers obtained in (trans)esterification of γ-PGA with poly(3HB-*co*-4HB) mediated by 4-toluenesulfonic acid monohydrate. Among proposed structure of γ-PGA-*graft*-(3HB-*co*-4HB) cooligomers, both linear and cyclic γ-PGA backbones were taken into consideration.

**Table 1:**
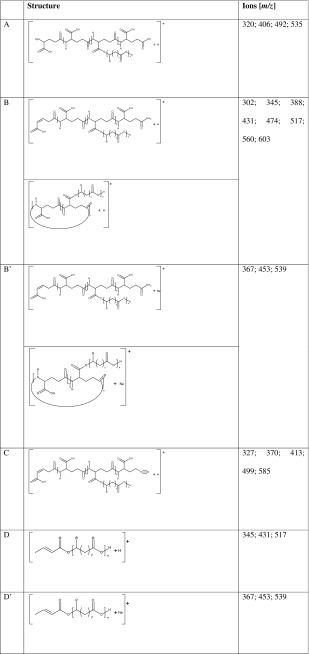
Structural Assignments of the Ions Appearing in the Expanded Regions at *m/z* 300–610 of the ESI-MS Spectrum presented in Figure 6

**Figure 7:**
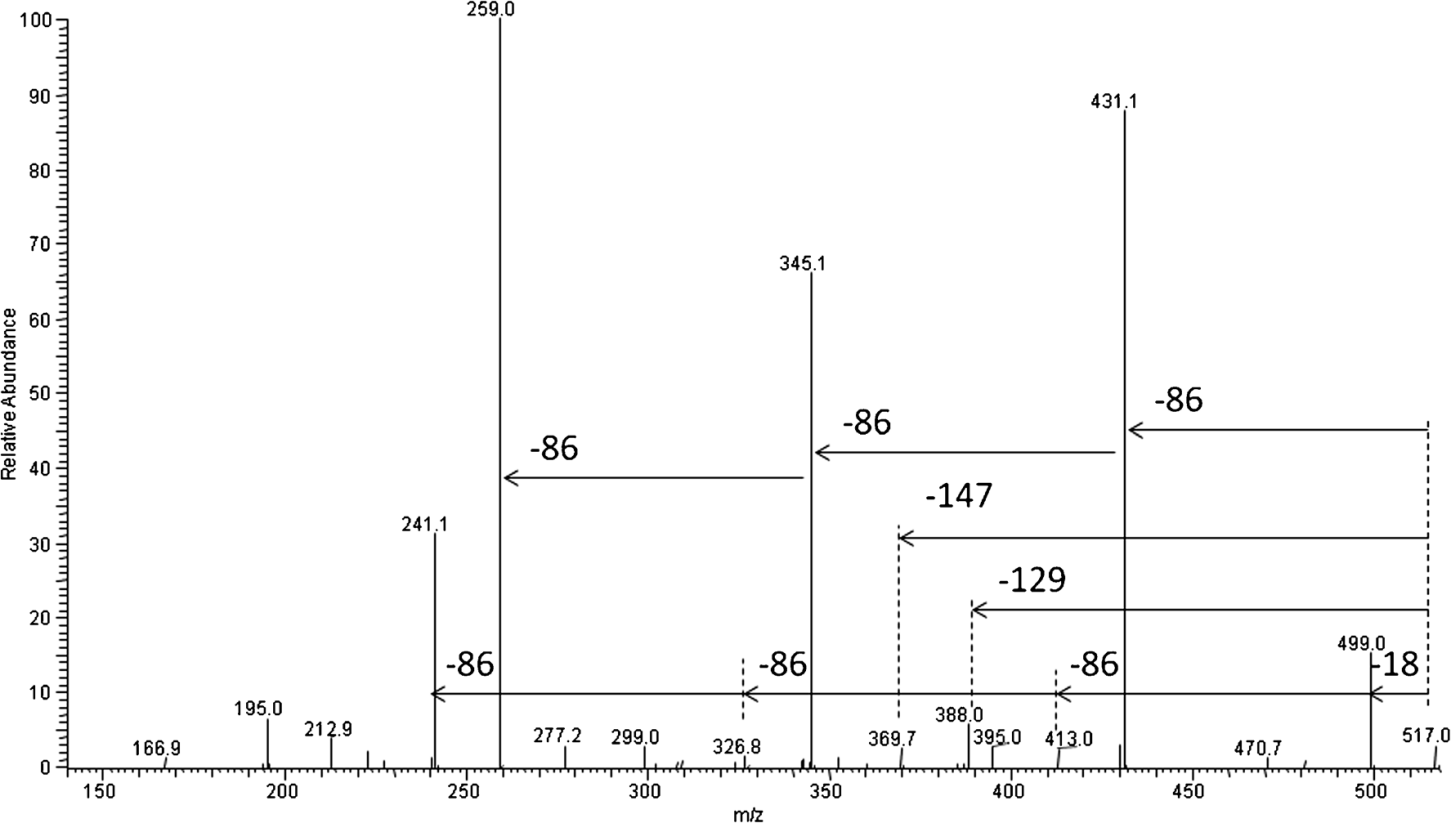
ESI-MS^2^ product ion spectrum of the sodiated γ-PGA-*graft*-(3HB-*co*-4HB) cooligomers at *m/z* 517 and theoretical fragmentation pathway of this ion

**Scheme 1:**
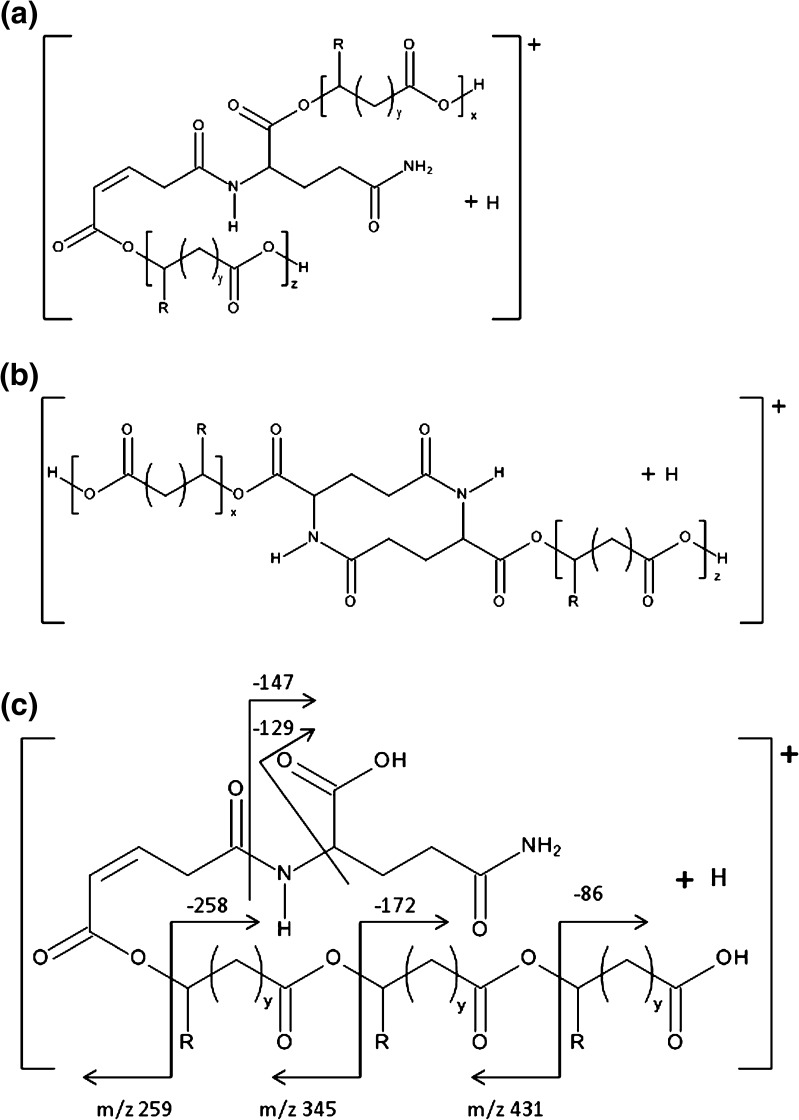
Probable structures of sodium adduct of γ-PGA-*graft*-(3HB-*co*-4HB) cooligomers at *m/z* 517 (x + z = 3; R = CH_3_, y = 1 for 3HB units; R = H, and y = 2 for 4HB units): linear (**a**), cyclic (**b**), and fragmentation pathway of one of possible structure of this ion (**c**)

As a part of structural analysis, the ESI-MS^2^ experiments for selected ions were carried out. Figure 7 shows the results of the ESI-MS^2^ experiment performed for the ion at *m/z* 517 selected from ESI-MS spectrum (labeled B, see structure in Table 1). This ion corresponds to sodium adduct of γ-PGA-*graft*-(3HB-*co*-4HB) cooligomers containing three repeating units derivative from poly(3-hydroxybutyrate-*co*-4-hydroxybutyrate) and two repeating units derivative from γ-PGA. The oligoamide part of such ion could be cyclic or might contain unsaturated alkyl and amide end groups (see Scheme 1). The product ion at *m/z* 370 corresponds to the cooligomer formed by the loss of the γ-glutamic acid (147 Da). The product ion at *m/z* 388 corresponds to the cooligomer formed by the loss of the pyroglutamic acid (129 Da). The product ion at *m/z* 499 corresponds to the cooligomer formed by the loss of the water molecule (18 Da). The product ions at *m/z* 431; 345; 259 correspond to the cooligomer formed by the loss of the one (86 Da), two (172 Da), and three (258) 3-hydroxybutyric or 4-hydroxybutyric repeating units.

## Results of Cytocompatibility Studies of γ-PGA-Graft-(3HB-*co*-4HB) Copolymer

The cell viability was not affected by γ-PGA-*graft*-(3HB-*co*-4HB) copolymer over the entire range of concentrations (Figure S1, Supplementary Material). These results indicate that the novel materials obtained via (trans)esterification reaction of the poly-γ-glutamic acid with poly(3-hydroxybutyrate-*co*-4-hydroxybutyrate) mediated by 4-toluenesulfonic acid monohydrate carried out in melt did not affect negatively the viability of the treated cells.

## Conclusions

Two methods of obtaining graft copolymers composed of poly-γ-glutamic acid backbone and oligoesters pendant chains were developed. The first elaborated synthetic strategy is based on the anionic grafting of racemic β-butyrolactone on γ-PGA backbone. The second developed method is based on the (trans)esterification reaction of γ-PGA with poly(3-hydroxybutyrate-*co*-4-hydroxybutyrate) mediated by 4-toluenesulfonic acid monohydrate.

The structural studies with the aid of electrospray ionization multistage mass spectrometry technique confirmed the structure of graft copolymers obtained via both methods. Moreover, it was established that fragmentation of selected sodium adducts of graft copolymers proceeded via random breakage of amide bonds along the backbone and ester bonds of the oligoesters pendant chains. The ESI-MS allowed determining a variety of end groups in final products in case of applying the second method. The presence of various end groups was attributable to hydrolysis and thermal degradation, both of which occurred under reaction conditions. However, obtained copolymers, despite having a variety of end groups, did not affect negatively the viability of the treated cells during in vitro cytotoxicity tests.

In addition, the ESI-MS technique has allowed us to monitor the progress of hydrolytic degradation process of obtained copolymers and to determine the degradation products. The performed tests confirmed that hydrolytic degradation of oligoesters pendant chains proceeds faster than hydrolytic degradation of γ-PGA backbone.

This first method of synthesis of graft copolymers will be further developed in order to obtain copolymers with higher molecular weight, which should have thermo-mechanical properties required to find application in the field of biomaterials.

## Electronic supplementary material

Below is the link to the electronic supplementary material.

ESM 1 (DOCX 63 kb)
