# Supplementary material for: The Synthesis and Structural Characterization of Graft Copolymers Composed of γ-PGA Backbone and Oligoesters Pendant Chains
Source: J Am Soc Mass Spectrom. 2017 Jul 10;28(10):2223–34. doi: 10.1007/s13361-017-1731-y (PMC5594058; doi:10.1007/s13361-017-1731-y)
Supplement: Supplementary file 1 — (DOCX 63 kb) [file 13361_2017_1731_MOESM1_ESM.docx]

Journal of the American Society for Mass Spectrometry

The synthesis and structural characterization of graft copolymers composed of γ-PGA backbone and oligoesters pendant chains

Iwona Kwiecień^1^*, Iza Radecka^2^, Marek Kowalczuk^1,2^, Katarzyna Jelonek^1^, Arkadiusz Orchel^3^, Grażyna Adamus^1^

^1^Centre of Polymer and Carbon Materials, Polish Academy of Sciences, M. Curie-Skłodowskiej 34 Street, 41-819 Zabrze, Poland
^2^School of Biology, Chemistry and Forensic Science, Faculty of Science and Engineering, University of Wolverhampton, Wulfruna Street, Wolverhampton WV1 1SB, UK

^3^School of Pharmacy with the Division of Laboratory Medicine in Sosnowiec, Medical University of Silesia, Katowice, Poland, Chair and Department of Biopharmacy, 8 Jednosci Street, 41-208 Sosnowiec, Poland

*Corresponding author, e-mail: ikwiecien@cmpw-pan.edu.pl tel: +48 32 2716077 ext. 217

**Synthesis of graft copolymers via “grafting from” method.**

The aqueous solution of tetradecyltrimethylammonium bromide was added dropwise to the γ-PGA solution in phosphate buffer and stirring under room temperature overnight. The white precipitate was isolated by centrifugation, washed three times with distilled water and freeze-dried for 72 h. Obtained macroinitiator was placed into a round bottom flask and dissolved in DMSO. After the β-butyrolactone was added, the reaction mixture was stirred under argon atmosphere at the room temperature. The progress of the reaction was monitored by Fourier transform infrared (FT-IR) spectroscopy based on the intensity of the signals of the carbonyl group corresponding to the β-butyrolactone at 1820 cm^–1^ and the carbonyl group corresponding the oligo(3-hydroxybutyrate) pendant chains at 1735 cm^–1^. When the monomer conversion achieved 100%, the cation-exchange resin was added and the reaction mixture was stirred vigorously for 2 hours. The cation-exchange resin was removed by filtration and reaction mixture was placed into dialysis tubes and dialyzed for 60 hours. Then solution from the dialysis bag was freeze-dried for 24h.





**Scheme S1**. The anionic grafting of racemic β-butyrolactone on γ-PGA backbone.

**Synthesis of graft copolymers via (trans)esterification reaction**

The high molecular weight poly-γ-glutamic acid (0.15 g), poly(3-hydroxybutyrate-*co*-4-hydroxybutyrate) (0.2 g) and 4-toluenesulfonic acid monohydrate (0.08 g; 40 wt. % compare to poly(3-hydroxybutyrate-*co*-4-hydroxybutyrate) were placed into a round bottom flask equipped with a magnetic stirring bar. The reaction was carried out in the melt at 177-182°C under an argon atmosphere for 2 minutes. Then the reaction mixture was cooled down to room temperature, dissolved in DMF and dialyzed through dialysis tubes for 60 hours. Precipitated products, settled at the bottom of the dialysis bag, were dried under vacuum at room temperature for 48 hours. The reaction yield reached 65%.





**Scheme S2**. The (trans)esterification reaction of poly-γ-glutamic acid with poly(3-hydroxybutyrate-*co*-4-hydroxybutyrate) in the presence of 4-toluenesulfonic acid monohydrate (for 3HB units R = CH_3_, y = 1, for 4HB units R = H and y = 2).

**Assessment of cytocompatibility of γ-PGA-graft-(3HB-*co*-4HB) copolymer**

*In vitro* cytotoxicity was analyzed after an indirect contact of CCD-11Lu fibroblasts with extracts of the γ-PGA-graft-(3HB-*co*-4HB) copolymer by means of sulforhodamine B based assay (“In Vitro Toxicology Assay Kit, Sulforhodamine B based”; Sigma-Aldrich). Extracts of the polymer samples were prepared in triplicate according to ISO 10993-12 at a ratio of 0.2 g of powdered sample / mL of cell culture medium at 37 °C for 72-h extraction period. The samples were centrifuged at 3500 rpm for 5 minutes and supernatant was used for cytotoxicity tests. A dilution series of original extracts in growth medium (1:4, 1:8, 1:16 and 1:32 ratios of extract to fresh medium) were prepared.  Cells treated with growth medium incubated at the same conditions as original extracts (diluted 1:4, 1:8, 1:16 and 1:32 ratios with fresh medium) were used as the appropriate controls. 5 % DMSO in culture medium was used as a positive control.

The normal human lung fibroblast cell line (CCD-11Lu) was obtained from ATCC (LGC Standards, Lomianki, Poland) and grown in complete culture medium containing DMEM (Dulbecco’s Modified Eagle’s Medium, Sigma-Aldrich) supplemented with 10% FBS (HyClone), 100U/mL penicillin, 100 µg/mL streptomycin (Sigma-Aldrich), and 20mM HEPES (Sigma-Aldrich). Both cell lines were maintained at 37°C in a humidified atmosphere containing 5% CO_2_.

To assess cell proliferation, fibroblasts were seeded into 96-well plates (3× 10^3^ cells/well in 200 μL of culture medium) and allowed to adhere for 24h. Subsequently, the medium was replaced with fresh medium containing increasing concentrations of the tested compounds, and the cells were cultured for the next three days. Each concentration was tested four times for each of three samples, and experiments were repeated at least three times.

At the end of the incubation period the medium was aspirated and the cells were fixed with 10% trichloroacetic acid, washed with deionized water and stained with 0.4% sulforhodamine B (SRB). After rinsing out the unincorporated dye using 1% acetic acid, the incorporated stain was solubilized in 200μL of 10 mM Tris solution. Absorbance was measured at 570 nm and 690 nm (reference wavelength) using the MRX Revelation plate reader (Dynex Technologies).

The data were analyzed using a one way ANOVA and statistical analysis was performed using ANOVA and Tukey’s test. All the results are expressed as means ± SD. 𝑃 value of <0.05 was considered statistically significant.

Cytotoxicity was evaluated using various dilutions of the γ-PGA-*graft*-(3HB-*co*-4HB) extracts. The extract dilution method is commonly adopted for the *in vitr*o cytotoxicity evaluation, because it can be applied to a wide variety of raw materials and final products. It allowed for the interaction of leachable compounds with the CCD-11Lu cells. The results were compared with cells treated with growth medium incubated at the same conditions as original extracts.





**Figure S1**. Effect of γ-PGA-graft-(3HB-*co*-4HB) on cellular viability (P < 0.05 versus the control group).
